# Associations between sleep hygiene and secondary objective sleep parameters: moving beyond sleep duration

**DOI:** 10.1007/s10865-026-00634-w

**Published:** 2026-03-19

**Authors:** Thomas McAlpine, Barbara A. Mullan, Patrick J. F. Clarke

**Affiliations:** 1 enAble Institute, Faculty of Health Sciences, Curtin University, Bentley, WA 6102 Australia; 2 School of Population Health, Faculty of Health Sciences, Curtin University, Bentley, Australia

**Keywords:** Sleep hygiene, Wake after sleep onset, Sleep onset latency, Number of awakenings, Linear mixed effects

## Abstract

The present study sought to better understand the relationship between a broad range of sleep hygiene behaviours and important but under researched objective sleep outcomes: total time spent awake after sleep onset (WASO), the number of awakenings (NWAK) during the night, and sleep onset latency (SOL). Secondary data from a 14-day repeated measures design among 74 undergraduate students was analysed using linear mixed models to determine the between and within-person associations of 32 sleep hygiene behaviours with objectively measured sleep outcomes. Later bedtimes and earlier wake-times were significantly associated with better sleep at both the between and within-person levels. More frequent napping was associated with increased NWAK and greater WASO. More frequent occurrences of falling asleep with music or the TV on, eating too much food before bed, and alcohol use were each only associated with an increased NWAK. Having an unpleasant conversation before bed and sleeping in a poorly ventilated environment were associated with greater WASO. Differential associations with sleep hygiene and individual sleep outcomes suggest that sleep hygiene could be reframed to incorporate sleep more holistically and more carefully consider which sleep parameter is being impacted.

**Supplementary Information:**

The online version contains supplementary material available at 10.1007/s10865-026-00634-w.

## Introduction

As a biological necessity for survival that has established effects on health and psychological wellbeing sufficient sleep is a critical goal for much of the global population (Mader et al., 2022). There are many components to achieving ‘good quality’ sleep including sufficient sleep length, falling asleep in an efficient manner, staying asleep after the first sleep onset, and limiting the number of awakenings during the night (Harvey et al., 2008). While clinical populations make up a large portion of those who experience inadequate sleep, insufficient sleep is also common in the broader population (Chattu et al., 2018), which has serious implications for a range of health, psychosocial, and economic outcomes (Bernert et al., 2014; Hillman et al., 2018; Hoevenaar-Blom et al., 2011; Rosekind et al., 2010). Given that many established sleep interventions focus specifically on treating mechanisms that maintain disordered sleep (e.g., Continuous Positive Airway Pressure therapy for obstructive sleep apnoea, CBT-I for insomnia; Rybarczyk et al., 2013; Uyumaz et al., 2021) such clinically-focused interventions may not be translatable for the general public. Instead, there is a need to focus on practices and habits that promote healthy sleep, referred to as sleep hygiene behaviours (De Pasquale et al., 2024). A comprehensive understanding of the role that sleep hygiene plays in improving and/or maintaining sleep quality in non-clinical presentations of disturbed sleep is critical to promoting healthy sleep in the general population.

While the concept of sleep hygiene is intuitively appealing as a pathway for improving sleep, there are several issues with its conceptualisation and use for non-clinical populations which limit the efficacy of sleep-promoting efforts which use sleep hygiene as primary components (De Pasquale et al., 2024). Part of the problem stems from a lack of consensus as to which behaviours are important for promoting sleep, evidenced by differing types and numbers of behaviours included in scales which range from 13 to 30 (Lacks & Rotert, 1986; Mastin et al., 2006; Yang et al., 2010). Furthermore, certain behaviours (e.g., napping, caffeine use) demonstrate inconsistent associations with sleep (Irish et al., 2015) with their impact on sleep still unclear. A reliance on cross-sectional methodologies may partially account for limited certainty regarding the directional influence of sleep hygiene behaviours on sleep, but even recent meta-analyses of experimental research have demonstrated negligible impacts on sleep for some behaviours like evening exercise (Frimpong et al., 2021; Stutz et al., 2019). Importantly, they suggest that behaviour can differentially affect sleep, potentially impacting some components negatively (sleep onset latency, sleep efficiency) but not others (slow wave sleep).

The acknowledgement of such inconsistent findings highlights the need to build a more robust evidence base on which practical recommendations can be adopted for general populations. Recent work focussing on caffeine, napping, and exercise has made important steps forward in highlighting that individual differences may be responsible for how certain behaviours may impact sleep. For instance, tolerance effects of caffeine may limit its sleep-disturbing properties for habitual users (Irish et al., 2021), and a person’s chronotype (preference for being a morning or evening person) appears to moderate the relationship between late exercise and sleep (Glavin et al., 2020). While such research improves our understanding, more needs to be done to further quantify the inconsistencies that are often observed with sleep hygiene (McAlpine et al., 2023a).

One aspect of the relationship between sleep hygiene behaviours and sleep that has been relatively overlooked is the type of sleep parameter considered. While it is well known that sleep is multi-faceted, much of the current research draws on correlations between the previous performance of sleep hygiene behaviours and single subjective measures of sleep (e.g., Brick et al., 2010; McAlpine et al., 2023b; Sancho-Domingo et al., 2020; Yang et al., 2010). As such, it is important to consider multiple indicators of sleep to gain a more complete picture of the ways in which sleep hygiene behaviours are associated with sleep (if at all).

Sleep parameters which are typically examined include sleep duration, given its clear and established link with many short and long-term health outcomes (Cappuccio et al., 2010; Hoevenaar-Blom et al., 2011; McAlpine et al., 2023a), or sleep efficiency (McAlpine et al., 2023a; Yan et al., 2021). However, other important parameters include wake after sleep onset (total time spent awake after first falling asleep), the number of awakenings that occur during a sleep period, and sleep onset latency (how long it takes to fall asleep). Such sleep parameters, while often overlooked, have been demonstrated to influence health over and above sleep duration (see Buysse, 2014) so should be considered as important and independent components of sleep too. Individuals are also not typically good at estimating these parameters (Kobayashi et al., 2012; Williams et al., 2020) and therefore objective measures of sleep may be best placed to assess them.

These aspects of sleep are comparatively under-researched but provide alternative explanatory avenues for how sleep hygiene may impact sleep. For instance, rumination type ‘behaviours’ which are often included in sleep hygiene scales (Mastin et al., 2006; Yang et al., 2010), are said to increase cognitive arousal, which is believed to contribute to a psychophysiological state incompatible with quality sleep. However, it may be that such behaviours affect sleep by delaying the sleep onset phase (Valck et al., 2004), rather than necessarily impacting on post sleep onset parameters (e.g., the efficiency of a sleep period obtained after finally falling asleep). Furthermore, other behaviours seem more likely to impact wakefulness after falling asleep (e.g., movements from partners or pets), rather than impacting sleep latency. Both these aspects of sleep contribute to sleep efficiency, an important component of sleep health (Buysse, 2014) which in turn is independently related to various adverse health outcomes (e.g., Didikoglu et al., 2020; Marques et al., 2017). Therefore, identifying which sleep hygiene behaviours may be disrupting these aspects of sleep health have downstream implications for public health.

The aim of the present study is therefore to explore the relationship of a wide range of sleep hygiene behaviours with sleep parameters that to the best of our knowledge have not been examined. Using secondary data from a previous study (McAlpine et al., 2023a) we sought to clarify the relationship between 32 sleep hygiene behaviours and three sleep outcomes; wake after sleep onset (WASO), the total number of awakenings (NWAK), and sleep onset latency (SOL). Due to the large number of behaviours explored, an exploratory approach was adopted rather than offering numerous hypotheses spanning multiple predictors and multiple outcomes.

## Materials and methods

### Procedure and participants

The present study uses secondary data drawn from a previous examination of 74 Australian university undergraduate students. A more detailed description of the procedure can be found in McAlpine et al. (2023a), however relevant details are summarised below. A 14-day repeated measures design was used to capture the day-to-day performance of 32 sleep hygiene behaviours and nightly sleep outcomes. Baseline induction sessions were followed by 14 nights of sleep data collected using accelerometry, and brief daily measures of sleep hygiene performance and subjective measures of sleep which were obtained at the participants specified typical wake-up time. No participants dropped out, and the extent of missingness was low, with 95.6% of all daily surveys completed and missing data ranging from 0 to 12.7% for each variable, totalling 4.5% missing data across all timepoints. Upon completion, participants were awarded course credit.

## Measures

*Sleep Outcomes.* Actigraph GT9X Link© (ActiGraph LLC., Pensacola, FL, USA) accelerometers were used to collect several sleep outcomes, of which three were used for the present study; SOL (the time it took participants to fall asleep at night), NWAK (total number of times that actigraphy recorded an awakening following sleep onset and before the final identified wake period), and WASO (the total amount of time in minutes that actigraphy determined was spent awake following sleep onset and before the final identified wake period). To calculate each of these parameters, the Cole-Kripke algorithm was applied to participant’s sleep periods. Accelerometers were set to record triaxial data at 30 Hz with an epoch value of 60 s.

Sleep periods were identified with the Tudor-Locke algorithm within the ActiLife (v6.13.4) software and in conjunction with the consensus sleep diary (Carney et al., 2012) were finalised by marrying sharp spikes/troughs in activity with self-reported sleep times, an approach that has been used in previous research to help validate actigraphy against gold standard estimates such as polysomnography (Quante et al., 2018). The consensus sleep diary contains several self-report questions about the previous night’s sleep, but of particular importance were the two items “What time did you try to go to sleep?” and “What time was your final awakening?“, which were used to validate sleep periods.

*Sleep Hygiene.* As per McAlpine et al. (2023b), the current study combined a widely inclusive range of sleep hygiene behaviours from validated sleep hygiene scales (Lacks & Rotert, 1986; Mastin et al., 2006; Yang et al., 2010) in a manner that eliminated redundancy (item overlap) while capturing the broad set of behaviours that potentially contribute to sleep. Ultimately, 32 sleep hygiene behaviours were assessed in the present study (Table 1).

*Demographic Variables.* Self-reported age, gender and prior diagnosis of sleep disorder were collected at baseline inductions to be used as control variables where relevant.

**Table 1:** List of sleep hygiene behaviours assessed

Sleep Hygiene Behaviour
Worrying about falling asleep while in bed
Having an unpleasant conversation before bed
Falling asleep with music or the TV on
Pondering unresolved matters before bed
Checking the time in the middle of the night
Worrying about sleep during the daytime
Engaging in vigorous intensity exercise (e.g., boxing, running > 5 mph / > 8 kph, swimming, soccer, jumping rope, etc.)
Napping during the day
Having any exposure to sunlight or outdoor light during the day
Engaging in moderate intensity exercise (e.g., briskly walking, lightly cycling, cleaning heavily, etc.)
Going to bed hungry
Going to bed thirsty
Having caffeine before bed
Consuming any other stimulating substances before bed (e.g., nicotine)
Having alcohol before bed
Drinking too much water before bed
Eating too much food before bed
Going to sleep in an environment that is too noisy (or quiet)
Going to sleep in an environment that is too bright (or dark)
Going to sleep in an environment that is too humid (or dry)
Going to sleep in an environment that is poorly ventilated
Going to sleep on an uncomfortable bed or pillow/s
Having sleep interrupted by a partner (e.g., snoring, changing positions)
Use a screen (phone, computer, laptop, TV, etc.) before bed (*IF YES*) - Using social media on this device (e.g., Facebook, Instagram, Snapchat, Twitter, Tik Tok)
Engaging in activities of high concentration before bed
Feeling stressed out or in another emotional state before bed (e.g., angry, upset)
Using sleep medications to help fall asleep
Drinking alcohol with the intention of using it to help fall asleep
Having sleep interrupted by pets
Using social media before bed
Going to bed at a different time than usual
Waking up at a different time than usual

### Statistical analyses

R and the jomo package (Quartagno & Carpenter, 2022) were used to perform multiple imputations which can account for the clustered nature of the data (i.e., correlated repeated measurements within individuals). From the initial dataset, ten more were imputed, each 1000 iterations apart after an initial burn-in phase of 5000 draws. Convergence was assumed after examination of the trace and autocorrelation plots.

To assess the relationship between sleep hygiene behaviours and two of the sleep outcomes (WASO and NWAK), the lme4 (Bates et al., 2015), nlme (Pinheiro et al., 2021), and mitml (Grund et al., 2021) packages were used to construct linear mixed models. Participants were fitted as a random factor in each model, and the following process was followed for each sleep outcome. Null models containing only the demographic variables were constructed to assess the association with each sleep outcome. Significant demographic predictors were retained in a base model which contained all sleep hygiene behaviours. Sleep hygiene behaviours were disaggregated into within and between effects before being added to the model in line with recommendations (Curran & Bauer, 2011). Between effects therefore represent the association of sleep parameters with the frequency (proportion) with which a sleep hygiene behaviour was performed over the 14 days (i.e., a frequency value of 1 represents daily performance whereas values closer to 0 represent infrequent performance). Between-person coefficients therefore represent the predicted difference in the outcome between someone who never performs the behaviour against someone who performs it daily. Likewise, within effects therefore represent the association of sleep parameters with the individual daily performance of sleep hygiene behaviours (i.e., the predicted effect of performing vs. not performing the behaviour on the sleep parameters of the subsequent sleep period). To help with model convergence, continuous variables were grand-mean standardised before being added into the SOL models.

Base models were compared to the null model to assess whether there was significant improvement in the fit using the D3 pooled likelihood ratio-test (Grund et al., 2021), which is appropriate when models have the same random effects structure. Significant behaviours are reported in their respective tables (Tables 3, 4 and 5). Individual random slopes models for each sleep hygiene behaviour were then fitted and compared to the base model to test whether the within-person effect of sleep hygiene behaviour on each sleep outcome significantly varied for individuals (i.e., if the within-person effect depends on the individual). As the random effects structures differ for these model comparisons, a likelihood ratio test with a boundary-corrected reference distribution was used (½χ²₀ + ½χ²₁; Self & Liang, 1987).

SOL was non-normally distributed, so generalised linear mixed models were adopted instead, with the Gamma family and the log link function applied to account for the exponential (decay) distribution. However, the analysis procedure remained largely the same as the other sleep outcomes.

## Results

### Descriptives

Demographic and descriptive information of the sample are shown in Table 2.

**Table 2:** Sociodemographic and sleep characteristics of the sample across 14 nights (N = 74)

Categorical Variables	*N* (%)
*Gender*	
Man	21 (28.4%)
Woman	52 (70.3%)
Non-binary	1 (1.4%)
*Ethnicity*	
Asian	20 (27.0%)
Caucasian	49 (66.2%)
Other	5 (6.8%)
*Education*	
High School	51 (68.9%)
Certificate I-IV	12 (16.2%)
Advanced Diploma/Diploma	3 (4.2%)
Bachelor’s Degree	7 (9.5%)
Graduate Certificate/Diploma	1 (1.4%)
*Children Under Two*	
Yes	2 (2.7%)
No	72 (97.3%)
*Self-Reported Sleep Disorder*	
None	70 (94.6%)
Insomnia	3 (4.1%)
Sleep Apnoea	1 (1.4%)

Continuous Variables	*M* (*SD*)
Age	22.12 (5.59)
Wake After Sleep Onset (mins)	90.00 (27.41)
Number of Awakenings (#)	28.78 (7.45)
Sleep Onset Latency (mins)	7.29 (7.19)
Total Sleep Time (mins)	393.68 (61.88)
Bedtime	11:59 pm (1:51)
Waketime	8:09 am (1:56)

### Wake after sleep onset

The null model was constructed and none of the demographic variables were significantly related to the outcome. As such, the base model with all predictor variables included showed significant improvements over the null model *(F* = 6.27, *df*_1_ = 60, *df*_2_ = 91247.40, *p* < .001). The base model showed several independent predictors of WASO (Table 3) at both the between and within-person level.^1^

**Table 3:** Full linear mixed model of wake after sleep onset being predicted by all sleep hygiene behaviours

Random effects	Group	Variance	SD	ICC
	Participant (intercept)	411.28	20.28	0.40
Fixed Effects (Between)	B	SE	t	*p*
(Intercept)	103.48	26.51	3.90	< 0.001
Bedtime***	– 45.44^†^	7.92	– 5.74	< 0.001
Waketime***	39.52^†^	7.44	5.31	< 0.001
Pondering Unresolved Matters	20.08	17.99	1.12	0.264
Exposure to Sunlight (during day)	– 28.41	17.68	– 1.61	0.108
Screen Use	– 11.07	30.10	– 0.37	0.713
High Concentration	– 18.33	16.42	– 1.12	0.264
Negative Emotional States	13.12	23.30	0.56	0.574
Night-time Worry (about sleep)	– 37.76	24.15	– 1.56	0.118
Unpleasant Conversation	3.88	44.45	0.09	0.930
Music or TV	23.07	12.15	1.90	0.058
Checking the Time (during sleep period)	6.96	18.29	0.38	0.704
Daytime Worry (about sleep)	11.16	23.21	0.48	0.631
Vigorous Exercise	– 2.56	16.83	– 0.15	0.879
Napping**	85.69	29.24	2.93	0.003
Moderate Exercise	– 21.41	14.92	– 1.43	0.152
Hungry	21.17	30.96	0.68	0.494
Thirsty	– 37.56	23.48	– 1.60	0.110
Caffeine*	– 37.95	18.42	– 2.06	0.040
Other Stimulating Substances	– 19.77	21.35	– 0.93	0.355
Alcohol	36.79	18.84	1.95	0.051
Too Much Water*	– 71.14	30.27	– 2.35	0.019
Too Much Food	49.07	35.53	1.38	0.167
Noisy or Quiet Environment	30.48	45.07	0.68	0.499
Bright or Dark Environment	– 1.37	43.01	– 0.03	0.975
Humid or Dry Environment	65.11	94.46	0.69	0.491
Poor Ventilation	89.22	91.88	0.97	0.332
Uncomfortable Bed Environment	– 4.09	34.18	– 0.12	0.905
Being Awoken by Partner	– 27.96	19.87	– 1.41	0.159
Social Media Use	26.54	14.92	1.78	0.076
Sleep Medications	– 9.70	15.53	– 0.62	0.532
Deliberately Using Alcohol to Help Sleep	– 33.85	34.79	– 0.97	0.331
Being Awoken by Pets	– 9.02	18.20	– 0.50	0.620

Fixed Effects (Within)	B	SE	t	*p*
Bedtime***	– 12.62^†^	0.87	– 14.45	< 0.001
Waketime***	12.62^†^	0.87	14.56	< 0.001
Pondering Unresolved Matters	3.81	2.57	1.48	0.140
Exposure to Sunlight (during day)	– 4.11	2.73	– 1.50	0.133
Screen Use	– 5.06	3.54	– 1.43	0.153
High Concentration	– 1.66	2.68	– 0.62	0.536
Negative Emotional States	2.19	2.58	0.85	0.396
Night– time Worry (about sleep)	2.33	3.13	0.74	0.458
Unpleasant Conversation*	9.36	4.59	2.04	0.042
Music or TV	3.72	3.38	1.10	0.270
Checking the Time (during sleep period)	1.99	2.64	0.75	0.452
Daytime Worry (about sleep)	– 1.50	5.21	– 0.29	0.774
Vigorous Exercise	– 0.44	3.06	– 0.14	0.886
Napping	– 4.88	3.42	– 1.43	0.154
Moderate Exercise	– 1.52	2.11	– 0.72	0.471
Hungry	– 0.16	4.48	– 0.04	0.972
Thirsty	– 2.87	3.46	– 0.83	0.406
Caffeine	1.62	3.22	0.50	0.614
Other Stimulating Substances	8.20	5.80	1.42	0.157
Alcohol	2.31	3.04	0.76	0.447
Too Much Water	– 3.28	4.43	– 0.74	0.459
Too Much Food	3.02	3.89	0.78	0.438
Noisy or Quiet Environment	– 1.87	5.06	– 0.37	0.713
Bright or Dark Environment	– 2.51	6.26	– 0.40	0.689
Humid or Dry Environment	11.48	9.35	1.23	0.220
Poor Ventilation*	18.51	8.93	2.07	0.038
Uncomfortable Bed Environment	5.00	5.84	0.86	0.392
Being Awoken by Partner	4.94	3.52	1.41	0.160
Social Media Use	0.49	3.03	0.16	0.872
Sleep Medications	– 0.22	14.45	– 0.02	0.988
Deliberately Using Alcohol to Help Sleep**	– 38.55	12.83	– 3.00	0.004
Being Awoken by Pets	4.27	3.46	1.23	0.218

ICC = Intraclass coefficient. SE = Standard Error

^†^ As continuous variables, bedtime and waketime were standardised before being entered into the model and coefficients therefore represent the change in the outcome associated with one standard deviation change in the predictor

* Denotes significance at the *p* < .05 level. ** *p* < .01. *** *p* < .001

At the between-persons level, later mean bedtimes, earlier mean waketimes, more frequent caffeine use, and more frequent occurrences of having too much water before bed were associated with less WASO. Furthermore, a greater frequency of daytime napping was significantly associated with more WASO.

At the within-person level, later than usual bedtimes, earlier than usual waketimes, and using alcohol with the specific intention of assisting with sleep were associated with less WASO. Furthermore, having an unpleasant conversation before bed and sleeping in a poorly ventilated environment were significantly associated with greater WASO.

Given these models contained many predictors, the potential for suppressor effects were considered. We therefore repeated the analyses with only the significant effects (Supplementary Table 5) included. The pattern of results did not change with the exception of the between-persons effect of having too much water before bed, which was no longer significant, although it should be noted that the effect size was comparable to other significant predictors.

Separate models with each sleep hygiene behaviour fitted as a random slope were also constructed. To help with convergence only one random slope was fitted at a time before being compared to the base model and this was repeated separately for each sleep hygiene variable. Significance for multiple hypothesis testing was therefore set at the Bonferroni corrected alpha level of 0.002 and only the addition of three random slopes showed significant improvements over the base model (random intercepts only model). That is, the within-person effect of going to bed, χ²(1, mixture) = 20.87, *p* < .001 or waking up, χ²(1, mixture) = 38.66, *p* < .001 at a different time to usual, and going to bed hungry, χ²(1, mixture) = 47.60, *p* < .001 varied significantly by participant.

### Number of awakenings

The null model was constructed and only age was significantly related to NWAK. Age was therefore retained in the base model which included all sleep hygiene variables and when compared to the null model, had significantly better fit *(F* = 6.74, *df*_1_ = 61, *df*_2_ = 29147.99, *p* < .001). The base model showed several independent predictors of NWAK (Table 4) at both the between and within-person level.^2^  

**Table 4:** Full linear mixed model of number of awakenings being predicted by all sleep hygiene behaviours

Random effects	Group	Variance	SD	ICC
	Participant (intercept)	11.39	3.38	0.23
Fixed Effects (Between)	B	SE	t	*p*
(Intercept)	45.11	5.44	8.30	< 0.001
Age***	– 0.61	0.13	– 4.81	< 0.001
Bedtime***	– 10.97^†^	1.43	– 7.68	< 0.001
Waketime***	8.77^†^	1.36	6.47	< 0.001
Pondering Unresolved Matters	4.51	3.22	1.40	0.162
Exposure to Sunlight (during day)	– 3.54	3.13	– 1.13	0.257
Screen Use	– 4.22	5.42	– 0.78	0.436
High Concentration	4.46	3.11	1.43	0.152
Negative Emotional States	– 4.09	4.22	– 0.97	0.332
Night– time Worry** (about sleep)	– 12.26	4.27	– 2.88	0.004
Unpleasant Conversation	2.08	8.21	0.25	0.800
Music or TV*	4.39	2.23	1.97	0.048
Checking the Time (during sleep period)	3.13	3.73	0.84	0.403
Daytime Worry (about sleep)	5.43	4.07	1.34	0.182
Vigorous Exercise	1.99	3.06	0.65	0.515
Napping**	16.30	5.29	3.08	0.002
Moderate Exercise	– 3.53	2.69	– 1.31	0.189
Hungry	6.88	5.72	1.20	0.230
Thirsty	– 7.48	4.28	– 1.75	0.081
Caffeine	– 2.05	3.35	– 0.61	0.540
Other Stimulating Substances	– 2.37	4.12	– 0.58	0.566
Alcohol*	7.94	3.52	2.26	0.024
Too Much Water	– 3.68	5.63	– 0.65	0.514
Too Much Food**	20.40	6.52	3.13	0.002
Noisy or Quiet Environment	10.37	8.63	1.20	0.230
Bright or Dark Environment	– 8.89	8.44	– 1.05	0.293
Humid or Dry Environment	14.27	17.53	0.81	0.416
Poor Ventilation	14.80	17.21	0.86	0.390
Uncomfortable Bed Environment	– 6.15	6.15	– 1.00	0.318
Being Awoken by Partner	– 1.69	3.50	– 0.48	0.630
Social Media Use	2.11	2.59	0.81	0.416
Sleep Medications	– 2.63	2.80	– 0.94	0.349
Deliberately Using Alcohol to Help Sleep	– 9.19	6.36	– 1.44	0.149
Being Awoken by Pets	– 5.90	3.43	– 1.72	0.086

Fixed Effects (Within)	B	SE	t	*p*
Bedtime*	– 3.72^†^	0.22	– 16.72	< 0.001
Waketime*	3.63^†^	0.23	15.73	< 0.001
Pondering Unresolved Matters	– 0.70	0.71	– 0.98	0.329
Exposure to Sunlight (during day)	– 0.43	0.76	– 0.57	0.566
Screen Use	– 0.83	0.91	– 0.91	0.365
High Concentration	0.37	0.68	0.55	0.582
Negative Emotional States	0.06	0.68	0.09	0.929
Night-time Worry (about sleep)	– 0.20	0.79	– 0.25	0.799
Unpleasant Conversation	0.48	1.24	0.38	0.702
Music or TV*	1.92	0.87	2.22	0.027
Checking the Time (during sleep period)	– 0.41	0.67	– 0.62	0.536
Daytime Worry (about sleep)	0.45	1.37	0.33	0.741
Vigorous Exercise	0.82	0.81	1.02	0.307
Napping	– 0.12	0.88	– 0.13	0.894
Moderate Exercise	0.32	0.58	0.55	0.582
Hungry	– 1.52	1.20	– 1.27	0.207
Thirsty**	– 2.42	0.88	– 2.77	0.006
Caffeine	0.01	0.80	0.01	0.993
Other Stimulating Substances	1.23	1.59	0.78	0.438
Alcohol	– 0.25	0.77	– 0.33	0.742
Too Much Water	0.44	1.11	0.40	0.690
Too Much Food	0.27	0.98	0.27	0.785
Noisy or Quiet Environment	– 0.71	1.31	– 0.54	0.587
Bright or Dark Environment	0.70	1.67	0.42	0.676
Humid or Dry Environment	– 0.03	2.33	– 0.01	0.989
Poor Ventilation	3.32	2.29	1.45	0.147
Uncomfortable Bed Environment	– 0.67	1.74	– 0.39	0.700
Being Awoken by Partner	– 1.00	0.90	– 1.11	0.266
Social Media Use	1.32	0.79	1.67	0.096
Sleep Medications	– 1.74	3.67	– 0.47	0.636
Deliberately Using Alcohol to Help Sleep	– 3.33	3.13	– 1.06	0.291
Being Awoken by Pets	0.71	0.88	0.81	0.418

ICC = Intraclass coefficient. SE = Standard Error

^†^As continuous variables, bedtime and waketime were standardised before being entered into the model and coefficients therefore represent the change in the outcome associated with one standard deviation change in the predictor

Denotes significance at the *p* < .05 level. ** *p* < .01. *** *p* < .001

At the between-person level, older age, later mean bedtime, earlier mean waketime, and more frequent occurrences of worrying about falling asleep were associated with fewer awakenings. Furthermore, more frequent occurrences of nights falling asleep while listening to music/watching TV, eating too much food before bed, alcohol use, and daytime napping were all associated with more awakenings during the sleep period.

There were fewer associations demonstrated at the within-persons level^3^, with a later than usual bedtime and an earlier than usual waketime again associated with fewer awakenings, as was going to bed thirsty. Like the between-persons effect, falling asleep while listening to music/watching TV was also associated with more awakenings during the sleep period.

Like with the WASO models, we also repeated the main analyses with only the significant effects (Supplementary Table 6) included. The pattern of results was largely the same as reported here, with the exception of the between-persons effect of listening to music/watching TV, and alcohol, which were both no longer significant. Unlike WASO however, none of the Bonferroni corrected models for NWAK supported a significantly improved fit over the base model when random slopes were added.

### Sleep onset latency

The distribution of sleep latency was non-normal which meant that linear mixed models could not be applied. Instead, generalised linear mixed models were used for SOL, using the gamma family (as data was exponentially distributed) and the log link function. Previous diagnosis of sleep disorder was the only covariate significantly related to SOL in the null model. The base model including all sleep hygiene variables was constructed but model complexity caused non-convergence. Therefore, individual models were constructed for each sleep hygiene behaviour which included previous sleep disorder diagnosis as a control variable and both between and within components for each sleep hygiene variable. Significance was assessed at the Bonferroni adjusted alpha level of 0.002. Table 5 shows all models and their associations with SOL and estimates are expressed on the log scale with respect to the outcome.

**Table 5:** Estimates for each generalised linear mixed model predicting Sleep Onset Latency, controlling for previous diagnosis of sleep disorder

Model	Component	Estimate	SE	t	*p*
Bedtime	Between	0.10	0.04	2.74	0.006
	Within	– 0.04	0.03	– 1.03	0.305
Waketime	Between*	0.15	0.04	4.03	< 0.001
	Within	0.01	0.03	0.42	0.673
Pondering Unresolved Matters	Between	0.32	0.16	1.98	0.048
	Within	0.08	0.10	0.88	0.382
Exposure to Sunlight (during day)	Between	– 0.45	0.19	– 2.19	0.028
	Within	– 0.02	0.11	– 0.22	0.827
Screen Use	Between	– 0.15	0.29	– 0.50	0.620
	Within	0.20	0.12	1.64	0.101
High Concentration	Between	– 0.14	0.18	– 0.79	0.431
	Within	– 0.02	0.11	– 0.16	0.874
Negative Emotional States	Between	0.23	0.18	1.30	0.192
	Within	0.04	0.09	0.41	0.682
Night-time Worry (about sleep)	Between	0.53	0.22	2.37	0.018
	Within	0.29	0.13	2.30	0.022
Unpleasant Conversation	Between	0.73	0.36	2.01	0.044
	Within	0.21	0.18	1.20	0.233
Music or TV	Between	0.16	0.16	0.99	0.325
	Within	0.08	0.15	0.55	0.584
Checking the Time (during sleep period)	Between	0.38	0.18	2.15	0.032
	Within	– 0.04	0.10	– 0.41	0.684
Daytime Worry (about sleep)	Between	0.10	0.23	0.42	0.672
	Within	0.01	0.18	0.08	0.936
Vigorous Exercise	Between	– 0.27	0.19	– 1.38	0.169
	Within	– 0.19	0.13	– 1.48	0.140
Napping	Between	0.66	0.35	1.87	0.062
	Within	0.12	0.13	0.94	0.350
Moderate Exercise	Between	– 0.12	0.18	– 0.66	0.507
	Within	0.10	0.09	1.12	0.264
Hungry	Between	0.07	0.29	0.25	0.802
	Within	0.06	0.18	0.36	0.719
Thirsty	Between	0.16	0.20	0.83	0.410
	Within	– 0.08	0.15	– 0.57	0.571
Caffeine	Between	– 0.40	0.19	– 2.12	0.034
	Within	– 0.12	0.14	– 0.92	0.361
Other Stimulating Substances	Between	0.19	0.16	1.20	0.231
	Within	– 0.28	0.24	– 1.15	0.250
Alcohol	Between	0.09	0.17	0.53	0.594
	Within	– 0.05	0.13	– 0.40	0.689
Too Much Water	Between	– 0.38	0.26	– 1.45	0.148
	Within	0.14	0.17	0.82	0.411
Too Much Food	Between	0.02	0.38	0.06	0.956
	Within	0.08	0.16	0.49	0.628
Noisy or Quiet Environment	Between	– 0.28	0.18	– 1.54	0.123
	Within	0.32	0.18	1.74	0.082
Bright or Dark Environment	Between	– 0.37	0.17	– 2.17	0.030
	Within	0.25	0.26	0.95	0.343
Humid or Dry Environment	Between	– 0.08	0.88	– 0.09	0.927
	Within	0.31	0.34	0.91	0.363
Poor Ventilation	Between	0.86	0.76	1.14	0.257
	Within	0.50	0.34	1.46	0.143
Uncomfortable Bed Environment	Between	0.07	0.42	0.16	0.874
	Within	– 0.02	0.22	– 0.09	0.929
Being Awoken by Partner	Between	– 0.09	0.24	– 0.37	0.713
	Within	0.09	0.14	0.65	0.517
Social Media Use	Between	0.12	0.13	0.91	0.362
	Within	0.10	0.10	0.98	0.329
Sleep Medications	Between	– 0.15	0.18	– 0.82	0.411
	Within	0.18	0.50	0.35	0.727
Deliberately Using Alcohol to Help Sleep	Between	0.81	0.36	2.27	0.023
	Within	0.38	0.49	0.78	0.440
Being Awoken by Pets	Between	0.33	0.20	1.66	0.098
	Within	0.14	0.15	0.93	0.355

SE = Standard Error

* Denotes significance at the Bonferroni-adjusted *p* < .002 level

Only the between-person effect of waketime was significantly related to sleep at the adjusted alpha level, however there were some substantially large effect sizes observed for some variables. For instance, the between person coefficient for sunlight suggests that getting sunlight or outdoor light exposure for each day of the two-week period compared to getting sunlight exposure for none of the days is associated with a 36% less SOL. Similarly, the within person coefficient for worrying about sleep at night suggests that worrying about sleep on any given night is associated with a 34% increase in SOL when compared to nights where no nighttime worry was experienced.

Random slopes were also fitted for each sleep hygiene behaviour model, however model convergence was not achieved in most cases so inferences could not be made about whether allowing effects to vary for each participant provided an improvement over models with random intercepts only.

## Discussion

The present findings demonstrate specific associations with several sleep hygiene behaviours and the three sleep outcomes that were assessed (WASO, NWAK, and SOL). Firstly, bedtime and waketime showed consistent associations with both WASO and NWAK at both the between and within-person level. This pattern suggests that those with later average bedtimes seem to wake less often and spend less time awake than those with earlier bedtimes. Conversely, those with earlier mean waketimes seem to experience similar benefits. This is in opposition to previous findings which demonstrate that later bedtimes are associated with negative sleep outcomes, specifically, shorter sleep duration and lower sleep efficiency (McAlpine et al., 2023a). An immediately obvious explanation for this finding could be that those with later bedtimes generally experience reduced total sleep periods, therefore limiting the opportunity for awakenings to accumulate over the course of the night. Yet sensitivity analyses showed that these relationships were upheld even after controlling for sleep duration. An alternative explanation for the unexpected associations between later bedtime and less WASO and NWAK could be due to an increase in the homeostatic sleep pressure which occurs the longer one goes without sleep (Ehlen et al., 2013), since sleep continuity improves with increasing homeostatic pressure (Dijk & Landolt, 2019).

Those with later average waketimes also experienced longer times taken to fall asleep, as waketime was the only between-person effect that was significantly related with SOL at the corrected alpha level. The temporal direction of this relationship is not entirely clear but based on previous research on chronotype and the similarly large but non-significant effect size for the bedtime-SOL relationship, it seems likely that these results reflect the generally poorer sleep characteristics of evening-types (Harfmann et al., 2020; McMahon et al., 2019).

These findings extend to the within-person level suggesting that not only are these associations present for later and earlier mean bedtimes when comparing one person to the next, they are present for later and earlier bedtimes with respect to an individual’s typical sleep schedule (i.e., a later than usual bedtime may result in fewer awakenings for the subsequent sleep period). In addition to this, both schedule-based behaviours varied significantly by participant meaning that the association was stronger for some than it was for others. This suggests that there are individual factors to consider here and that perhaps wakefulness is more susceptible to the influence of bedtime scheduling for some people than for others. Although not assessed in the present research, future research might seek to explore what these potential individual factors are. For instance, does chronotype, or a preference for routine determine whether sleep schedule is associated with sleep outcomes?

Expectedly, and in line with other research assessing nap frequency in college students (Ye et al., 2015), more frequent nappers over the course of the 14 days were more likely to experience negative sleep outcomes. Specifically, more frequent daytime napping was associated with both more WASO, and with a greater NWAK. This adds to the literature which shows the acute associations of napping on same-night sleep (Mead et al., 2022) extend in such a way whereby those with more frequent napping habits are also at risk for a more disturbed sleep architecture. It should be noted however, that given the bidirectional nature of this relationship that is often observed (Häusler et al., 2019), the findings here could also represent those with poorer sleep being more likely to use naps as a compensatory mechanism, particularly since there were no significant within-persons effects for napping identified in the present study.

Younger age, more frequent use of music or TV while falling asleep, more frequent consumption of alcohol, and more frequent occasions of eating too much food before bed were similarly associated with more awakenings at night, however these associations were not present for total WASO. While the detrimental association of more frequent alcohol use with NWAK aligns with previous work exploring its negative association with sleep duration (Chaput et al., 2012; McAlpine et al., 2023a), neither music/TV use nor too much food have been associated with either sleep duration or efficiency at the between-person level (McAlpine et al., 2023a). This begins to highlight some of the differential associations between sleep hygiene behaviours and different sleep outcomes (i.e., some behaviours are correlated with specific components of sleep, but not others).

Beyond just frequent performance of behaviour, within-person effects demonstrated that performance of several sleep hygiene behaviours were acutely associated with subsequently impaired sleep with respect to both NWAK and WASO. Having an unpleasant conversation before bed and going to sleep in a poorly ventilated environment were both significantly associated with greater WASO. Both of these behaviours have been associated with shorter sleep duration and with poorer sleep quality (McAlpine et al., 2023a; Strøm-Tejsen et al., 2016), so the present findings extend on current understandings by demonstrating associations with additional sleep outcomes, suggesting that these two behaviours may influence multiple aspects of sleep. Furthermore, specific occasions of going to bed listening to music or watching TV was also associated with a greater NWAK at the within-person level. This finding seems to be supported by other research suggesting that TV use before bed is negatively associated with certain sleep outcomes (Exelmans et al., 2019). In fact, Exelmans et al. (2019) measured both habitual TV use and procrastinatory TV use and results were very similar to those demonstrated here, in that both habitual TV use and procrastinatory TV use were related to at least one negative sleep outcome, but not all. Consequently, it could be that the within-person effect demonstrated here is reflective of TV use in a procrastinatory manner. It should be noted however that this seems to be the first study to report specifically on the relationship between this variable and NWAK at night and that it conjunctly assesses music use in the same item. Given the complicated nature of the media and sleep relationship (Ellithorpe et al., 2022), and the many different ways in which media can be used, future research should consider treating these two behaviours as distinctly different, to perhaps best capture whether it is being used in a procrastinatory manner, or to help aid sleep instead (e.g., relaxing music before bed; Harmat et al., 2008).

Interestingly there were also several behaviours that were unexpectedly associated with beneficial sleep outcomes. More frequent use of caffeine and more frequent occurrences of having too much water before bed were associated with less WASO, whereas more frequent worry about falling asleep at night was associated with fewer total NWAK. For caffeine and too much water, it is important to recognise these as between-persons effects. As such, the associations do not imply that acute daily instances of either behaviour result in impaired sleep. Instead, they suggest that more frequent consumers of both generally tend to experience better sleep outcomes. The same pattern of findings were observed by McAlpine et al. (2023a) for a different sleep outcome whereby both of these behaviours were also associated with longer sleep duration. Leaning on their explanation, it therefore seems likely the between-effect observed here represents a generally increased level of water consumption being beneficial for sleep (Grandner et al., 2010), and for caffeine, may represent the more frequent (and therefore more habitual) users overcoming withdrawal symptoms to receive an overall net benefit. However, more research is needed to both replicate this finding in other populations and understand how and why habitual caffeine users might experience generally improved sleep outcomes when compared to less habitual users.

The association between worrying about sleep and decreased waking in the night seems counter-intuitive, as this has consistently been associated with poorer sleep outcomes (Clancy et al., 2020). Yet Sharman et al. (2022) also demonstrated that pre-cognitive arousal was not related to objectively measured periods of wake and it is possible that those that are more likely to worry before bed experience difficulties in one facet of sleep (onset) but not in others (e.g., maintenance) as sleep difficulties do not necessarily span both (Bollu & Kaur, 2019). This is somewhat supported with the effect sizes from the SOL analyses which showed that acute instances of nighttime worry were associated with a 33% increased SOL, although this association was not significant after correcting for multiple hypothesis testing.

Finally, two more behaviours exhibited within-person associations that infer a positive impact on sleep. Using alcohol with the intention of aiding sleep was associated with reduced WASO and going to bed thirsty was associated with fewer awakenings. Intentional alcohol use is likely to be result of the dual-natured impact of alcohol on sleep, whereby it has been shown to improve sleep in the first half of the sleep period but impair sleep in the second half (Arnedt et al., 2011). Furthermore, alcohol use in this manner may underly more dependent users for whom such use is often cited as a reason to help combat impaired sleep from withdrawal (Landolt & Gillin, 2001; Reid-Varley et al., 2020) and therefore reflects an acute improvement in sleep when used in such a way. As for going to bed thirsty, it is unclear why this behaviour was associated with fewer awakenings and research in this field is scant, so empirical explanations cannot be offered. However, going to bed thirsty implies a reduced need to use the bathroom at night so a logical explanation could be that going to bed thirsty simply reduces the number of times that someone may awake to relieve themselves, but more research is needed to confirm this.

### Strengths, limitations, and future directions

Although there were several strengths to the present research, there were also limitations worth noting. Repeated measures data collected in such a way allowed for a naturalistic observation of participants day-to-day behaviour. Coupled with the analytical procedure, this allowed for both between and within-person associations to be drawn from a very likely scenario whereby many sleep hygiene behaviours would be performed and therefore competing for their influence on sleep. As such, significant associations observed, controlled for the associations of all others and identified those that are most likely to uniquely impact sleep. Conversely, it is important to consider that given the broad range of behaviours, some may share variance in the outcomes and there may be some behaviours which overlap and influence sleep through the same mechanism (e.g., use of screen before bed and high levels of concentration might conjunctly increase cognitive arousal). To overcome this, future work might seek to build on previous identification of set sleep factors (McAlpine et al., 2023b) instead of numerous individual behaviours.

Another limitation was the SOL analysis, which after adjusting the alpha level demonstrated only one significant association. While we choose not to elaborate too much on non-significant findings, the trends observed reflected several associations that would be consistent with expectations. For instance, many of the behaviours that exhibited trends (pondering unresolved matters, night-time worry about sleep, checking the time at night, having an unpleasant conversation) are indicative of perseverative cognition and should be associated with SOL given the role that such cognitions play in insomnia (Harvey, 2002). It should also be noted that although wrist-worn actigraphy is generally accepted as providing reasonable estimates for sleep against gold standard measures such as polysomnography (Full et al., 2018; Marino et al., 2013), this can vary by the device and the specific parameter being assessed (Plekhanova et al., 2023) and further research could seek to replicate these findings across other measures of objective sleep.

A final limitation relates to the use of self-reported diagnosis of sleep disorders. Given the overall poorer sleep characteristics of the student population (Peltzer & Pengpid, 2016) - which was reflected in these results - there is a risk of undiagnosed or unrecognised disordered sleep in this sample. However, we controlled for self-reported sleep disorder in the analyses where it was related to the outcome being assessed, and since the vast majority of participants reported no sleep disorder, this risk is unlikely to have influenced the results.

## Conclusions

In combination with previous work (e.g., McAlpine et al., 2023a), the present findings critically highlight that the relationship between sleep hygiene behaviours and sleep must be considered holistically. Across sleep duration, sleep efficiency, SOL, WASO, and NWAK, some behaviours were only associated with just one sleep outcome, and only the timing of sleep appear to be associated with all. This should flag to researchers and practitioners alike that the mechanisms by which these behaviours affect sleep ought to be considered. When making recommendations to improve sleep via changes in sleep hygiene behaviours, care should be taken with either (a) how we are assuming these behaviours might improve sleep, and (b) for whom these behaviours affect sleep. It makes little sense for the lay person presenting solely with sleep latency problems to suggest specific behaviour changes that may only influence a different sleep outcome. Furthermore, arduously attempting to change, constrain, monitor and/or alter behaviour across a broad spectrum of potentially redundant behaviours minimises the effort-reward ratio and could limit the efficacy of such behaviour change efforts in improving sleep. While more research is needed to consolidate cross-sectional associations observed here (i.e., experimental manipulation), takeaways from the present research include that sleep hygiene interventions may need to be tailored to the specific needs of individuals to maximise the improvements that are observed.

## Supplementary Information

Below is the link to the electronic supplementary material.

Supplementary Material 1

## Data Availability

The data underlying this article cannot be shared publicly due to the conditions under which ethics were obtained. Participants were informed that only “The following people will have access to the information we collect in this research: the research team and, in the event of an audit or investigation, staff from the Curtin University Office of Research and Development”. As such we are unable to make data publicly available.

## Footnotes

1 We also considered that the possible influence of these predictors on WASO might be conflated by restricted sleep periods (i.e., less sleep duration should relate to less WASO), so sensitivity analyses were conducted with actigraphy-assessed sleep duration as a control variable. The results were very similar and there was no substantive difference to the pattern of results, so we retained the initial base model.

2 Like with the WASO model we considered that restricted sleep periods could be influencing the relationships between the predictors and NWAK, so sensitivity analyses were conducted with actigraphy-assessed sleep duration as a control variable. The results were very similar and there was no substantive difference to the pattern of results, so we retained the initial base model.

3 Given the low variance reported in some of the predictors we conducted post-hoc power simulations to determine whether power was an issue. Deliberate use of alcohol for sleep, and poor ventilation were two predictors that were potentially underpowered to predict the effect sizes reported (see Supplementary Material 4).
