# Supplementary material for: Associations between sleep hygiene and secondary objective sleep parameters: moving beyond sleep duration
Source: J Behav Med. 2026 Mar 19;49(3):514–28. doi: 10.1007/s10865-026-00634-w (PMC13303661; doi:10.1007/s10865-026-00634-w)
Supplement: Supplementary file 1 — Supplementary Material 1 [file 10865_2026_634_MOESM1_ESM.docx]

# Supplementary Materials

## Supplementary Material 1: Bias and Levels of Agreement Between Actigraphy and Consensus Sleep Diary Estimates

Repeated measures correlations were obtained using the rmcorr package in r (Bakdash & Marusich, 2017) to determine the within-person concordance between the actigraphy estimates and consensus sleep diary estimates. The consensus sleep diary does not directly ask participants to report the total time spent awake after sleep onset (WASO), so the repeated measures correlations are reported only for NWAK and SOL.

Results showed that although actigraphy and consensus sleep diary estimates were significantly correlated for both measures (NWAK, SOL), the strength of the association were both weak suggesting low concordance.

*Supplementary Table 1. Repeated Measures Correlation Between Actigraphy and Consensus Sleep Diary*

| **Variable** | ***r*_rm_** | **95% CI (LL, UL]** | ***p*** |
| --- | --- | --- | --- |
| NWAK | .06 | [.00, .13] | .037 |
| SOL | .21 | [.15, .27] | < .001 |

## Supplementary Material 2: Random Slopes Model Comparison Using Boundary-Corrected Mixture Method Across 10 Multiply Imputed Datasets

*Supplementary Table 2. Improvement in WASO Model Fit by Addition of Random Slopes as Assessed by Boundary Corrected Mixture Method Across 10 Multiply Imputed Datasets*

| **Variable** | **LRT Value Mean (SD)** | **LRT Range  [Min, Max]** | ***p* value Mean** | ***P* value Range [Min, Max]** |
| --- | --- | --- | --- | --- |
| Bedtime | 20.87 (1.72) | 17.70, 23.27 | < .001 | < .001, < .001 |
| Waketime | 38.66 (3.27) | 34.84, 44.67 | < .001 | < .001, < .001 |
| Pondering Unresolved Matters | 0.03 (0.09) | 0.00, 0.27 | .471 | .299, .500 |
| Exposure to Sunlight (during day) | 0.09 (0.27) | 0.00, 0.85 | .465 | .178, .500 |
| Screen Use | 1.82 (0.77) | 0.93, 3.37 | .100 | .033, .168 |
| High Concentration | N/A |  |  |  |
| Negative Emotional States | 2.31 (0.70) | 0.83, 3.09 | .073 | .039, .182 |
| Night-time Worry (about sleep) | 0.97 (0.40) | 0.56, 1.73 | .170 | .094, .228 |
| Unpleasant Conversation | 0.67 (0.45) | 0.05, 1.40 | .234 | .119, .411 |
| Music or TV | 2.18 (1.15) | 0.76, 4.20 | .090 | .020, .192 |
| Checking the Time (during sleep period) | 7.42 (1.06) | 5.96, 9.42 | .003 | .001, .007 |
| Daytime Worry (about sleep) | 0.00 (0.00) | 0.00, 0.01 | .497 | .469, .500 |
| Vigorous Exercise | 0.00 (0.01) | 0.00, 0.02 | .490 | .451, 0.500 |
| Napping | 3.83 (1.61) | 2.45, 6.52 | .035 | .005, .059 |
| Moderate Exercise | N/A |  |  |  |
| Hungry | 47.60 (2.44) | 44.90, 51.80 | < .001 | < .001, < .001 |
| Thirsty | 0.12 (0.20) | 0.00, 0.65 | .410 | .210, .500 |
| Caffeine | 0.34 (0.40) | 0.00, 1.00 | .336 | .158, .500 |
| Other Stimulating Substances | 0.04 (0.07) | 0.00, 0.21 | .450 | .322, .500 |
| Alcohol | N/A | N/A | N/A | N/A |
| Too Much Water | N/A | N/A | N/A | N/A |
| Too Much Food | N/A | N/A | N/A | N/A |
| Noisy or Quiet Environment | N/A | N/A | N/A | N/A |
| Bright or Dark Environment | 0.02 (0.05) | 0.00, 0.16 | .485 | .347, .500 |
| Humid or Dry Environment | 2.29 (0.65) | 1.10, 3.24 | .072 | .036, .147 |
| Poor Ventilation | 4.32 (1.26) | 2.09, 6.30 | .025 | .006, .074 |
| Uncomfortable Bed Environment | 0.29 (0.42) | 0.00, 1.30 | .353 | .127, .500 |
| Being Awoken by Partner | 0.25 (0.33) | 0.00, 1.05 | .363 | .153, .500 |
| Social Media Use | 0.55 (0.51) | 0.00, 1.66 | .265 | .099, .500 |
| Sleep Medications | 0.04 (0.09) | 0.00, 0.28 | .469 | .299, .500 |
| Deliberately Using Alcohol to Help Sleep | 0.41 (0.87) | 0.00, 2.15 | .415 | .071, .500 |
| Being Awoken by Pets | 2.11 (0.74) | 0.96, 3.12 | .083 | .039, .163 |

Note. N/A = Variance could not be estimated due to singular fit.

*Supplementary Table 3. Improvement in NWAK Model Fit by Addition of Random Slopes as Assessed by Boundary Corrected Mixture Method Across 10 Multiply Imputed Datasets*

| **Variable** | **LRT Value Mean (SD)** | **LRT Range  [Min, Max]** | ***p* value Mean** | ***P* value Range [Min, Max]** |
| --- | --- | --- | --- | --- |
| Bedtime | 6.20 (0.86) | 5.39, 7.90 | .007 | .002, .010 |
| Waketime | 6.91 (1.74) | 3.93, 9.97 | .007 | .001, .024 |
| Pondering Unresolved Matters | 0.11 (0.28) | 0.00, 0.90 | .443 | .171, .500 |
| Exposure to Sunlight (during day) | N/A | N/A | N/A | N/A |
| Screen Use | 4.35 (2.42) | 1.37, 10.25 | .035 | .001, .121 |
| High Concentration | 0.06 (0.17) | 0.00, 0.54 | .461 | .231, .500 |
| Negative Emotional States | 0.22 (0.47) | 0.00, 1.29 | .428 | .128, .500 |
| Night-time Worry (about sleep) | 2.21 (1.29) | 0.33, 4.21 | .099 | .020, .283 |
| Unpleasant Conversation | 0.15 (0.23) | 0.00, 0.72 | .389 | .198, .500 |
| Music or TV | 3.16 (1.57) | 1.10, 5.89 | .057 | .008, .147 |
| Checking the Time (during sleep period) | 0.26 (0.46) | 0.00, 1.48 | .380 | .112, .500 |
| Daytime Worry (about sleep) | 0.53 (1.56) | 0.00, 4.98 | .420 | .013, .500 |
| Vigorous Exercise | 0.00 (0.00) | 0.00, 0.00 | .500 | .500, .500 |
| Napping | 0.04 (0.12) | 0.00, 0.37 | .477 | .270, .500 |
| Moderate Exercise | 0.34 (0.27) | 0.00, 0.80 | .313 | .186, .500 |
| Hungry | 1.03 (1.64) | 0.00, 5.18 | .268 | .011, .500 |
| Thirsty | 0.53 (0.66) | 0.00, 2.20 | .289 | .069, .500 |
| Caffeine | N/A |  |  |  |
| Other Stimulating Substances | 0.16 (0.27) | 0.00, 0.74 | .401 | .194, .500 |
| Alcohol | 0.01 (0.02) | 0.00, 0.05 | .491 | .409, .500 |
| Too Much Water | 0.10 (0.31) | 0.00, 0.99 | .466 | .160, .500 |
| Too Much Food | 0.07 (0.13) | 0.00, 0.37 | .441 | .273, .500 |
| Noisy or Quiet Environment | 1.69 (1.02) | 0.55, 3.74 | .121 | .026, .229 |
| Bright or Dark Environment | 0.79 (0.66) | 0.00, 1.83 | .240 | .088, .500 |
| Humid or Dry Environment | 2.46 (0.59) | 1.51, 3.43 | .062 | .032, .110 |
| Poor Ventilation | N/A |  |  |  |
| Uncomfortable Bed Environment | 1.31 (1.31) | 0.00, 3.24 | .202 | .036, .485 |
| Being Awoken by Partner | 2.50 (1.68) | 0.95, 5.98 | .085 | .007, .165 |
| Social Media Use | 0.40 (0.94) | 0.00, 2.99 | .393 | .042, .500 |
| Sleep Medications | 0.28 (0.45) | 0.00, 1.34 | .375 | .124, .500 |
| Deliberately Using Alcohol to Help Sleep | 5.67 (3.11) | 1.22, 11.91 | .027 | .000, .134 |
| Being Awoken by Pets | 0.03 (0.08) | 0.00, 0.27 | .480 | .303, .500 |

Note. N/A = Variance could not be estimated due to singular fit.

*Supplementary Table 4. Improvement in SOL Model Fit by Addition of Random Slopes as Assessed by Boundary Corrected Mixture Method Across 10 Multiply Imputed Datasets*

| **Variable** | **LRT Value Mean (SD)** | **LRT Range  [Min, Max]** | ***p* value Mean** | ***P* value Range [Min, Max]** |
| --- | --- | --- | --- | --- |
| Bedtime | 5.62 (1.01) | 4.48, 7.67 | .010 | .003, .017 |
| Waketime | 4.14 (1.86) | 2.38, 7.66 | .031 | .003, .061 |
| Pondering Unresolved Matters | 5.67 (3.07) | 2.75, 13.50 | .017 | < .001, .049 |
| Exposure to Sunlight (during day) | 7.45 (3.73) | 3.94, 17.22 | .007 | < .001, .024 |
| Screen Use | 4.78 (1.21) | 2.93, 6.31 | .018 | .006, .044 |
| High Concentration | 2.81 (1.75) | 0.27, 6.25 | .085 | .006, .300 |
| Negative Emotional States | 8.02 (3.79) | 4.87, 15.30 | .006 | < .001, .014 |
| Night-time Worry (about sleep) | 13.12 (4.76) | 7.18, 22.43 | .001 | < .001, .004 |
| Unpleasant Conversation | 3.97 (1.91) | 1.58, 7.71 | .038 | .003, .104 |
| Music or TV | 2.56 (0.97) | 0.87, 3.84 | .067 | .025, .175 |
| Checking the Time (during sleep period) | 0.98 (0.38) | 0.39, 1.57 | .171 | .105, .266 |
| Daytime Worry (about sleep) | 1.77 (1.47) | 0.54, 4.26 | .135 | .020, .231 |
| Vigorous Exercise | 3.65 (1.30) | 1.33, 6.00 | .038 | .007, .124 |
| Napping | 6.86 (1.80) | 4.29, 9.83 | .007 | .001, .019 |
| Moderate Exercise | 6.10 (2.18) | 3.10, 10.81 | .012 | .001, .039 |
| Hungry | 1.39 (0.84) | 0.01, 2.91 | .157 | .044, .463 |
| Thirsty | 2.70 (2.17) | 0.70, 7.97 | .086 | .002, .201 |
| Caffeine | 2.08 (1.30) | 0.46, 4.07 | .109 | .022, .249 |
| Other Stimulating Substances | 3.61 (1.73) | 0.70, 6.96 | .049 | .004, .201 |
| Alcohol | 0.49 (0.34) | 0.06, 1.24 | .262 | .133, .406 |
| Too Much Water | 2.97 (0.89) | 1.65, 4.33 | .049 | .019, .099 |
| Too Much Food | 1.12 (1.10) | 0.04, 3.51 | .205 | .030, .423 |
| Noisy or Quiet Environment | 5.14 (0.84) | 4.04, 7.18 | .013 | .004, .022 |
| Bright or Dark Environment | 2.20 (0.73) | 0.98, 3.33 | .078 | .034, .161 |
| Humid or Dry Environment | 1.25 (0.44) | 0.86, 2.15 | .139 | .071, .177 |
| Poor Ventilation | 7.22 (2.04) | 4.59, 11.46 | .006 | < .001, .016 |
| Uncomfortable Bed Environment | 0.41 (0.35) | 0.07, 1.03 | .286 | .155, .395 |
| Being Awoken by Partner | 2.30 (1.19) | 1.01, 4.31 | .084 | .019, .157 |
| Social Media Use | 3.11 (0.94) | 1.55, 4.35 | .046 | .019, .107 |
| Sleep Medications | 1.03 (1.14) | 0.02, 3.31 | .226 | .035, .440 |
| Deliberately Using Alcohol to Help Sleep | 2.27 (1.55) | 0.54, 4.87 | .101 | .014, .231 |
| Being Awoken by Pets | 6.45 (3.47) | 3.68, 12.82 | .014 | <.001, .028 |

## Supplementary Material 3: Condensed Models with Significant Predictors Only

*Supplementary Table 5. Simplified WASO Model*

| ***Random Effects*** |  |  |  |  |
| --- | --- | --- | --- | --- |
|  | ***Group*** | ***Variance*** | ***SD*** | ***ICC*** |
|  | *Participant (intercept)* | *656.97* | *25.63* | *.51* |
| ***Fixed Effects (Between)*** |  |  |  |  |
|  | ***B*** | ***SE*** | ***t*** | ***p*** |
| *(Intercept)* | *89.2* | *4.52* | *19.92* | *< .001* |
| *Bedtime** | *-37.44^†^* | *6.21* | *-6.03* | *< .001* |
| *Waketime** | *35.59^†^* | *6.14* | *5.80* | *< .001* |
| *Napping** | *80.75* | *31.47* | *2.57* | *.011* |
| *Caffeine** | *-35.63* | *15.84* | *-2.25* | *.024* |
| *Too Much Water** | *-34.17* | *21.60* | *-1.58* | *.114* |
| ***Fixed Effects (Within)*** |  |  |  |  |
|  | ***B*** | ***SE*** | ***t*** | ***P*** |
| *Bedtime** | *-12.87^†^* | *0.85* | *-15.19* | *< .001* |
| *Waketime** | *12.78^†^* | *0.85* | *15.00* | *< .001* |
| *Unpleasant Conversation** | *11.48* | *4.40* | *2.65* | *.008* |
| *Poor Ventilation** | *22.13* | *8.44* | *2.62* | *.009* |
| *Deliberately Using Alcohol to Help Sleep** | *-37.53* | *12.59* | *-2.98* | *.004* |

*Note*. ICC = Intraclass coefficient. SE = Standard Error.
^†^As continuous variables, bedtime and waketime were standardised before being entered into the model and therefore coefficient therefore represents the change in the outcome associated with one standard deviation change in the predictor.

*Supplementary Table 6. Simplified NWAK Model*

| ***Random Effects*** |  |  |  |  |
| --- | --- | --- | --- | --- |
|  | ***Group*** | ***Variance*** | ***SD*** | ***ICC*** |
|  | *Participant (intercept)* | *17.44* | *4.18* | *.31* |
| ***Fixed Effects (Between)*** |  |  |  |  |
|  | ***B*** | ***SE*** | ***t*** | ***p*** |
| *(Intercept)* | *38.85* | *2.37* | *16.38* | *< .001* |
| *Age** | *-0.54* | *0.11* | *-4.94* | *< .001* |
| *Bedtime** | *-8.94^†^* | *1.09* | *-8.21* | *< .001* |
| *Waketime** | *7.34* | *1.09* | *6.72* | *< .001* |
| *Night-time Worry* (about sleep)* | *-8.41* | *3.18* | *-2.65* | *.008* |
| *Music or TV** | *1.75* | *2.09* | *0.84* | *.403* |
| *Napping** | *13.92* | *5.24* | *2.66* | *.008* |
| *Alcohol** | *3.34* | *2.51* | *1.33* | *.183* |
| *Too Much Food** | *14.89* | *5.17* | *2.88* | *.004* |
| ***Fixed Effects (Within)*** |  |  |  |  |
|  | ***B*** | ***SE*** | ***t*** | ***p*** |
| *Bedtime** | *-3.74^†^* | *0.22* | *-17.39* | *< .001* |
| *Waketime** | *3.62^†^* | *0.22* | *16.16* | *< .001* |
| *Music or TV** | *1.90* | *0.85* | *2.24* | *.026* |
| *Thirsty** | *-2.23* | *0.87* | *-2.58* | *.010* |

*Note*. ICC = Intraclass coefficient. SE = Standard Error.
^†^As continuous variables, bedtime and waketime were standardised before being entered into the model and therefore coefficient therefore represents the change in the outcome associated with one standard deviation change in the predictor.

## Supplementary Material 4: Post-hoc Power Simulations

Given there were significant associations identified across multiple models at both the between and within-person level, there was clearly power to detect effects for *some predictors*. However, in these multi-level models, power is also influenced by variance, so it is possible that while we were powered for some predictors, we were underpowered for others. To get a practical estimate of the point in which we could be confident that we were appropriately powered to detect effects, we modelled the point at which variance at the within person level would be too small to detect a practically significant effect size. That is the point at which low variance resulted in less than 80% power to detect an effect size (β = 0.30), in a sample of 74 students with 14 nights of data. This was an arbitrary value, but this corresponds to approximately 10 minutes of extra WASO a night and 2 or more extra awakenings which we deemed was a practically meaningful and noticeable effect.

*Supplementary Figure 1. Within Person Variance Needed for Sufficient Power to Detect an Effect of* β = 0.30 *or Larger.*

*
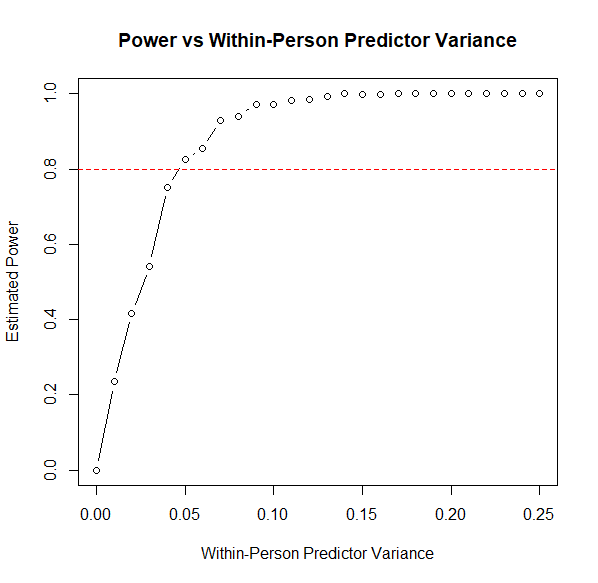
*

Results showed that within-person predictor variance would need to be at least 0.05 to detect an effect of this size or greater with 80% power. We then cross referenced the actual variance of each predictor in our sample which suggests that several predictors (noted in supplementary table 7) may have been underpowered. To confirm this further, we then cross-referenced against the reported effect sizes in the model to see if β ≥ 0.30. None of the predictors that were underpowered had effect sizes of at least this large in the WASO model. However, for NWAK, poor ventilation and deliberate use of alcohol experienced large enough but non-significant effect sizes and represent potentially underpowered predictors in this model.

*Supplementary Table 7. Reported Within Person Variance Across Each Predictor*

| **Predictor** | **Variance** |
| --- | --- |
| Pondering Unresolved Matters | 0.08 |
| Exposure to Sunlight (during day) ^†^ | 0.01 |
| Screen Use^†^ | 0.02 |
| High Concentration | 0.07 |
| Negative Emotional States | 0.10 |
| Night-time Worry (about sleep) ^†^ | 0.03 |
| Unpleasant Conversation | 0.13 |
| Music or TV^†^ | 0.05 |
| Checking the Time (during sleep period) ^†^ | 0.01 |
| Daytime Worry (about sleep) ^†^ | 0.04 |
| Vigorous Exercise | 0.16 |
| Napping | 0.06 |
| Moderate Exercise | 0.06 |
| Hungry | 0.08 |
| Thirsty^†^ | 0.03 |
| Caffeine | 0.06 |
| Other Stimulating Substances | 0.06 |
| Alcohol | 0.07 |
| Too Much Water^†^ | 0.00 |
| Too Much Food | 0.11 |
| Noisy or Quiet Environment^†^ | 0.02 |
| Bright or Dark Environment | 0.10 |
| Humid or Dry Environment | 0.06 |
| Poor Ventilation^†^ | 0.02 |
| Uncomfortable Bed Environment^†^ | 0.04 |
| Being Awoken by Partner | 0.15 |
| Social Media Use^†^ | 0.01 |
| Sleep Medications | 0.07 |
| Deliberately Using Alcohol to Help Sleep^†^ | 0.03 |
| Being Awoken by Pets | 0.08 |

^†^Predictors are likely to be underpowered to detect an effect of β ≤ 0.30.

**References**

Bakdash, J. Z., & Marusich, L. R. (2017). Repeated Measures Correlation [Methods]. *Frontiers in Psychology*, *8*, 1-14. <https://doi.org/10.3389/fpsyg.2017.00456>
